# Comparative Genomics of *Actinobacillus pleuropneumoniae* Serotype 8 Reveals the Importance of Prophages in the Genetic Variability of the Species

**DOI:** 10.1155/2020/9354204

**Published:** 2020-02-18

**Authors:** Isabelle Gonçalves de Oliveira Prado, Giarlã Cunha da Silva, Josicelli Souza Crispim, Pedro Marcus Pereira Vidigal, Moysés Nascimento, Mateus Ferreira Santana, Denise Mara Soares Bazzolli

**Affiliations:** ^1^Laboratório de Genética Molecular de Bactérias/Bioagro-Departamento de Microbiologia, Universidade Federal de Viçosa, Viçosa 36570-900, Brazil; ^2^Núcleo de Análise de Biomoléculas (NuBioMol), Universidade Federal de Viçosa, Viçosa, Brazil; ^3^Departamento de Estatística, Universidade Federal de Viçosa, Viçosa, Brazil

## Abstract

*Actinobacillus pleuropneumoniae* is the etiologic agent of porcine pleuropneumonia. Currently, there are 18 different serotypes; the serotype 8 is the most widely distributed in the United States, Canada, United Kingdom, and southeastern Brazil. In this study, genomes of seven *A. pleuropneumoniae* serotype 8 clinical isolates were compared to the other genomes of twelve serotypes. The analyses of serotype 8 genomes resulted in a set of 2352 protein-coding sequences. Of these sequences, 76.6% are present in all serotypes, 18.5% are shared with some serotypes, and 4.9% were differential. This differential portion was characterized as a series of hypothetical and regulatory protein sequences: mobile element sequence. Synteny analysis demonstrated possible events of gene recombination and acquisition by horizontal gene transfer (HGT) in this species. A total of 30 sequences related to prophages were identified in the genomes. These sequences represented 0.3 to 3.5% of the genome of the strains analyzed, and 16 of them contained complete prophages. Similarity analysis between complete prophage sequences evidenced a possible HGT with species belonging to the family Pasteurellaceae. Thus, mobile genetic elements, such as prophages, are important components of the differential portion of the *A. pleuropneumoniae* genome and demonstrate a central role in the evolution of the species. This study represents the first study done to understand the genome of *A*. *pleuropneumoniae* serotype 8.

## 1. Introduction

Pork is an important source of animal protein and is currently one of the most commonly consumed meat products in the world [1]. However, the use of an intensive production system has frequently given rise to the occurrence of respiratory diseases, having a major impact on production, causing significant economic losses in pig farming [2, 3]. Porcine pleuropneumonia is one of the most important respiratory diseases in pigs and is caused by the bacterium *Actinobacillus pleuropneumoniae*; this species can be divided into two biotypes according to their dependence on nicotinamide adenine dinucleotide (NAD) [4]. Currently, this species is classified into 18 serotypes based on the antigenic properties of capsule polysaccharides [5–7].

The pathogenesis of porcine pleuropneumonia is complex and involves different virulence factors produced by the bacterium [8–10]. Virulence is multifactorial and is related to a combination of factors such as toxins from the RTX family, composition and structure of capsule polysaccharides, outer membrane lipopolysaccharide (LPS), iron siderophores, biofilm formation, and adhesins [8]. In addition to the abovementioned virulence factors, some *A. pleuropneumoniae* serotypes present natural competence, and therefore, the occurrence of natural transformation is common in this species and dissemination of resistance genes in Pasteurellaceae family members, as *A. pleuropneumoniae*, is common [11–13]. Finally, virulence is complex, and antimicrobial resistance genes that can be encoded by both the chromosome and plasmids are essential depending on the specific niche, as in the natural hosts and in specific conditions [14, 15].

Although comparative genomic studies with different genotypes of *A. pleuropneumoniae* serotypes were carried out [16], no information on *A. pleuropneumoniae* serotype 8 was provided so far. Over the years, *A. pleuropneumoniae* serotype 8 has been neglected in identification studies due to failures in serotyping techniques, and as a result, genomic studies involving this serotype are nonexistent. Although recent studies have showed a wide distribution of this serotype in several regions, such as the United Kingdom [17, 18], North America [19], and Brazil [20], just recently *A. pleuropneumoniae* serotype 8 genome sequence was available [21, 22].

A study carried out by our research group using the alternative host, *Galleria mellonella* larvae, detected different virulence patterns in clinical isolates of serotype 8 *A. pleuropneumoniae* [23]. Based on the results obtained from that study, six isolates with different phenotypic profiles were selected for genomic sequencing. Clinical isolates from *A. pleuropneumoniae* serotype 8 have virulence complexity [9], but no specific information on genotypic variability is available so far. Then, this study is the first to describe *A. pleuropneumoniae* serotype 8 genomic from comparative analysis between *A. pleuropneumoniae* serotype 8 genomes (clinical isolates: Brazilian origin [21], one of English origin [22]) and twelve genomes of different serotypes of *A. pleuropneumoniae* deposited in databases.

## 2. Materials and Methods

### 2.1. *Actinobacillus pleuropneumoniae* Genome Sequences

Nineteen genomes from different serotypes of *A. pleuropneumoniae* available at the GenBank database (https://www.ncbi.nlm.nih.gov/genbank/) were used in the present study. The genomes of *A. pleuropneumoniae* serotypes 1 (4074), 13 (JL03), 5 (L20), 7 (AP76), and 8 (MIDG2331) are closed. The genomes of the other serotypes are in contigs (Table 1).

### 2.2. Determination of Protein-Coding Sequence Set in *A. pleuropneumoniae* Serotype 8

The set of clusters of the coding DNA sequences (CDS) predicted for *A. pleuropneumoniae* serotype 8 was based on the seven genomes of clinical isolates taken from pig farms, six from Brazil [21] and one from the United Kingdom [22]. In this analysis, the CD-HIT v.4.6.1 program [24, 25] was used to consider an identity threshold of 0.85 to cluster the CDS. For the functional annotation of the *A. pleuropneumoniae* serotype 8 reference genome, five databases were used: COG [26], CDD [27], PFAM [28], SMART [29], and UNIPROT [30]. The similarity searches were carried out using the BLAST algorithm [31] considering an *E* value ≤ 10^−5^.

### 2.3. Comparative Analysis of Predicted CDS of Serotype 8 and the Other 12 Different Serotypes of *A. pleuropneumoniae*

In this analysis, 12 genomes of *A. pleuropneumoniae* different serotypes (1, 2, 3, 4, 5b, 6, 7, 9, 10, 11, 12, and 13) were used (Table 1). These sequences are deposited in the UNIPROT database [30]. The comparative analysis was carried out using the BLAST algorithm [31], contrasting the genomes of the serotypes analyzed against the *A. pleuropneumoniae* serotype 8 reference assembled in this study.

### 2.4. Analysis of *A. pleuropneumoniae* Orthologous Gene Groups

From the predicted CDS of the 12 different serotypes and 7 serotype 8 *A. pleuropneumoniae* genomes, a database containing 28002 CDS corresponding to all serotypes of the species was assembled. Using the CD-HIT v.4.6.1 program [24, 25], with an identity threshold of 0.70 identity to cluster the sequences, an analysis was carried out to characterize the total set of CDS of the species. The CD-HIT was used for clustering the sequence and for reducing redundancy among them, to improve the results.

The groups of CDS identified by the CD-HIT were classified as core, shared, or differential. Additionally, the predicted protein sequences of *A. pleuropneumoniae* serotype 8 were individually compared to the predicted protein sequences of the other serotypes using the BLAST algorithm [31].

### 2.5. Genome-Wide Analysis of Preferential Codon Usage and GC%

The analysis of the preferential use of codons and GC content was carried out using the EMBOSS program [32] for the different serotype genomes of *A. pleuropneumoniae*. The use of each synonymous codon was determined by calculating the RSCU (Relative Synonymous Codon Usage). The RSCU value calculated for each codon was the parameter used to evaluate the codon selection type, with values = 1 characteristic of codons used with equal frequency; values > 1 were positive selection and <1 negative selection.

### 2.6. Synteny Analysis

The analyses were derived from the closed genomes of *A. pleuropneumoniae* serotypes 03 (JL03), 5b (L20), 7 (AP76), and 8 (MIDG2331) and from the six genomes of *A. pleuropneumoniae* serotype 8 isolates of Brazilian origin (MV460, MV518, MV597, MV780, MV1022, and MV5651). Multiple alignments of the sequences of the *A. pleuropneumoniae* genomes were derived from the Progressive Mauve v.2.3.1 software program [33].

### 2.7. Analysis of Sequences Similar to Prophages

Sequences similar to the prophages present in all the *A. pleuropneumoniae* genomes used in this study were obtained through the PHASTER program [34]. The prophage sequences were aligned by MAFFT [35], and the alignment was edited using the GBLOCKS program [36]. A dendrogram using the Neighbor-Joining genetic distance grouping method was generated by the MEGA 6 program [37] with a bootstrap containing 2000 replicates. Prophage complete sequences were compared using BLAST [31] against the GenBank databases to identify possible horizontal gene transfers between bacteria. For this, a coverage and identity above 70% and an *E* value less than 10^−5^ were used as cutoff points. After editing, the alignment was obtained using the GBLOCKS program. Using the same complete sequences of prophages, an alignment was done with the Clustal Omega [38]. From the values of the identity matrix provided, a heat map was constructed with software R under version 3.5.1.

## 3. Results

### 3.1. Genomic Analysis of *A. pleuropneumoniae* Serotype 8

The total set of predicted CDS of *A. pleuropneumoniae* serotype 8 generated from the seven clinical isolates corresponded to 2352 sequences (Table 2). Of these, 1801 (76.6%) were considered core, 436 (18.5%) were shared with other serotypes, though not all, and 115 (4.9%) were predicted to be differential to *A. pleuropneumoniae* serotype 8 genomes. Among the 2352 CDS of *A. pleuropneumoniae* serotype 8, 1925 (81.8%) were categorized into the COG database. Among these were 1542 (80.1%) encode proteins with known functional categories (excluding “Unknown function” and “Prediction of general functions”).

From the distinction of the core, shared and differential regions of *A. pleuropneumoniae* serotype 8 CDS and clusters of ortholog groups were analyzed. Of the 1801 sequences comprising the core portion, 1685 were affiliated to the categories of the COG database. Among these, 1358 sequences (80.6%) represent known functional categories (Table 2). The majority of sequences characterized as core are related to amino acid metabolism and transport; ribosomal translation, structure, and biogenesis processes; biogenesis of the wall, membrane, and cell envelope; and production and conservation of energy, among other activities considered essential to the survival of the pathogen (Table 2).

As regards the shared portion, of the 436 sequences, 220 were affiliated with the COG database categories, of which 166 (75.5%) were known functional categories. Most sequences are related to the metabolism and transport of inorganic ions; biogenesis of the cellular envelope; replication, recombination, and DNA repair; and metabolism in general (Table 2; Supplementary Data [Supplementary-material supplementary-material-1]).

The differential portion of the *A. pleuropneumoniae* serotype 8 genomes showed 115 CDS. Only 20 sequences were affiliated to the COG database categories, of which 18 (90.0%) were known functional categories (Table 2). These differential C are related to the regulatory processes and HGT mechanisms such as plasmids and prophages (Table 2; Supplementary Data [Supplementary-material supplementary-material-1]). In this portion, CDS related to resistance to antibiotics such as tetracycline and florfenicol genes, transcriptional regulators such as LysR, DNA repair protein, transposon gamma-delta resolvase, transport proteins such as sodium and glutamate symmetric acetyltransferase, and prophage-related protein-coding sequences were reported (Table 2).

### 3.2. The Pangenome of *A. pleuropneumoniae*

From the 2984 clusters obtained from the total set of CDS of the species, the general characterization of the CDS with the distinction of the core, shared, and differential portions was carried out. Of the total, 1737 clusters were characterized as core region, present in the thirteen serotypes analyzed; 756 were clusters of CDS of shared proteins, and 491 clusters corresponded to CDS of differential proteins of each serotype. As regards the total genome of each serotype, the core portion averaged 82.5% (Table 3) showing conservation among the different serotypes.

### 3.3. Similarity Analysis between the Amino Acid Sequences Predicted for *A. pleuropneumoniae* Serotype 8 and Other Serotypes

**Figure 1:**
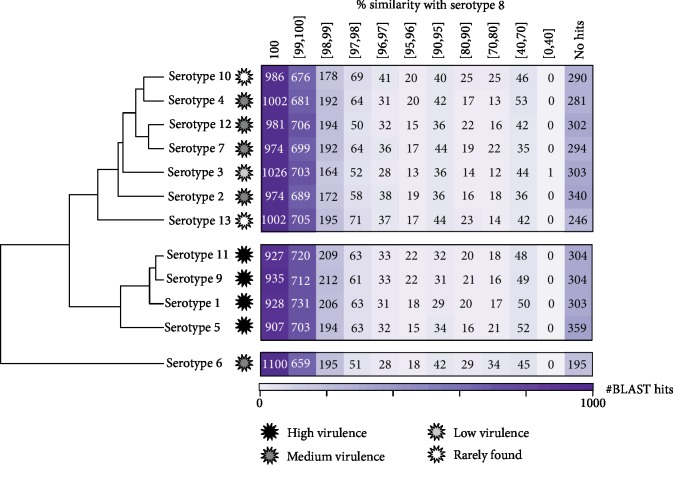
Analysis of similarity between predicted amino acid sequences of *A. pleuropneumoniae* serotype 8 and the other serotypes. The protein-coding sequences were clustered according to similarity in percentages to serotype 8. The serotypes were also characterized in relation to virulence.

An alignment between predicted amino acid sequences of *A. pleuropneumoniae* serotype 8 was created against all other *A. pleuropneumoniae* amino acid sequences used in this study generating clusters based on the pattern of similarity (Figure 1). Of the total 2352 amino acid sequences, 2196 (93.4%) had similarity patterns higher than 95%, thus revealing high serotype 8 sequence conservation in relation to the others. Based on the analysis of the BLAST results, three main groups of similarity related to high, medium, and low virulence standards were obtained. There was a greater sharing of the predicted CDS of serotype 8 with the serotype 6 sequences, followed by serotype 3 (Figure 1).

### 3.4. Codon Preferential Usage

**Figure 2:**
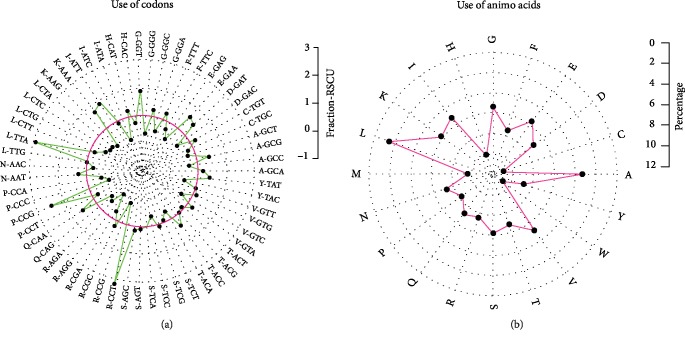
Use of codons and their respective amino acids of *A. pleuropneumoniae*. (a) The trend in the use of codons is represented in the circular map. Methionine, tryptophan, and stop codons were omitted. Synonymous codons for an amino acid used with equal frequency have RSCU = 1, indicated by the red circular line. (b) Percentage of the use of amino acids represented in the circular map. A: alanine; C: cysteine; D: aspartic acid; E: glutamic acid; F: phenylalanine; G: glycine; H: histidine; I: isoleucine; K: lysine; L: leucine; M: methionine; N: asparagine; P: proline; Q: glutamine; R: arginine; S: serine; T: threonine; V: valine; W: tryptophan; Y: tyrosine.

As regards the codon analysis, a high standard of conservation was observed in the use of codons among all *A. pleuropneumoniae* serotypes investigated, which includes the clinical isolates of serotype 8 analyzed in this study. In Figure 2, we have represented the use of codons by *A. pleuropneumoniae*. Codons with higher RSCU values result in higher positive selection for their respective amino acids (Figure 2). We observed no significant differences in the proportions of the use of amino acids between the different isolates nor between the serotypes. The most commonly used amino acids were leucine (L: 10.6%), alanine (A: 8.7%), isoleucine (I: 6.8%), and valine (V: 6.8%), while cysteine (C: 1.0%) and tryptophan (W: 1.2%) were the most rarely used (Figure 2).

### 3.5. Synteny Analysis of *A. pleuropneumoniae*

**Figure 3:**
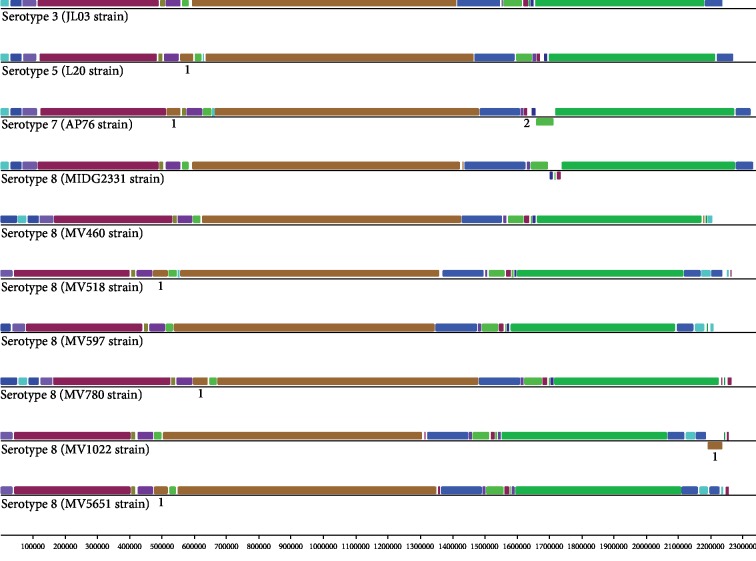
Synteny analysis of *A. pleuropneumoniae* genomes. The genomes represented correspond to serotype 3 (JL03 strain), serotype 5 (L20 strain), serotype 7 (AP76), and serotype 8 (MIDG2331, MV460, MV518, MV597, MV780, MV1022, and MV5651 strains). Horizontal bars represent the size of the genome (kb). The region identified in 1 represents the acquisition and loss of genomic information, and region 2 represents a recombination event.

In the alignment between the genotype-representative contigs of the six Brazilian clinical isolates of *A. pleuropneumoniae* serotype 8 and closed genomes of serotypes 3 (JL03), 5b (L20), 7 (AP76), and 8 (MIDG2331), we verified conservation in the genome structure (Figure 3). The genomes of *A. pleuropneumoniae* share practically the same blocks, denominated LCBs: “Selecting Locally Collinear Blocks.” Although genome alignment conservation was found, it was possible to observe regions of acquisition/loss of genetic material and rearrangements (Figure 3). The presence of a differential and conserved block in the genomes of serotypes 5 (L20) and 7 (AP76) as well as in four of the clinical isolates of serotype 8 (MV518, MV780, MV1022, and MV5651) was observed (Figure 3). In this block composed of approximately 42.206 pb and with GC content of 40.4%, there are sequences encoding proteins related to integrase (WP_005620278.1), pyrophosphatase (WP_005620280.1), RdgC recombination protein (WP_011848390.1), DNA methyltransferase (WP_011848391.1), antirepressor (WP_011848395.1, WP_011848414.1), DNA methylase (WP_011848397.1), endodeoxyribonuclease RuvA (WP_011848399.1), terminase (WP_043880767.1), peptidase (WP_043877971.1), and various phage proteins (WP_011848407.1, WP_011848405.1, WP_011848408.1, and WP_011848412.1). In a second differential genomic segment, present only in serotype 7, a region of inversion and rearrangement of a block of approximately 59.649 bp and GC content of 40.9% was present (Figure 3). In this segment, we found sequences corresponding to genes encoding carboxylase enzymes (WP_005617934.1, WP_005602033.1), oxidoreductases (WP_005598646.1, WP_005602054.1, and WP_012478542.1), reductase (WP_005598644.1), virulence factors involved in iron uptake (WP_005602070.1), integrase (WP_005617888.1), and transposases (WP_005599960.1).

Minor variations were also observed between the different genomes (Figure 3). Among these differences, we found a small region of approximately 13000 pb present only in the seven genomes of *A. pleuropneumoniae* serotype 8 and the CDS found were for tRNA-glutamate ligase (WP_005608501.1), tRNA-Ala (WP_005612726.1), preprotein translocase (WP_005612726.1), transcriptional regulator of the Rha family (WP_039768145.1), antirepressor (WP_058230489.1), propanediol utilization protein (WP_039768152.1), host death prevention protein family (Phd) (WP_005598318.1), YoeB toxin (WP_005605064.1), tetracyl disaccharide kinase (WP_005598320.1, WP_005608502.1), and nine sequences encoding hypothetical proteins (WP_039709488.1, WP_039768147.1, WP_039709486.1, WP_039709484.1, WP_039709483.1, WP_052250595.1, WP_014991324.1, WP_039768150.1, and WP_014991326.1). Another region of approximately 13180 pb was found in the genomes of *A. pleuropneumoniae* serotype 5 (L20), serotype 7 (AP76), and in three serotype 8 clinical isolates (MIDG2331, MV1022, and MV518). Sequences corresponding to genes present in this region were encoded for *flp*C operon (WP_039709641.1), *flp*B operon (WP_039709034.1, WP_011848427.1), fimbriae protein (WP_058230512.1, WP_039709035.1, WP_005611759.1, and WP_011848428.1), ATP-dependent ATP-RhI-RNA (WP_039709036.1), ATP-binding protein (WP_005611764.1), iron-ABC transport substrate binding (WP_005611765.1), and three hypothetical proteins (WP_005611761.1, WP_009875478.1, and WP_005611761.1).

### 3.6. Analysis of Prophage Sequences

30 sequences similar to the prophages were found in the 19 strains of *A. pleuropneumoniae* analyzed. From the total of sequences similar to prophages, 16 were classified by the PHASTER program as complete, 11 as incomplete, and 3 as questionable (Supplementary Data [Supplementary-material supplementary-material-1]). Incomplete and questionable sequences were considered genomic regions containing sequences derived from phages. The regions containing prophage-related genes represent 0.3 to 3.5% of the genomes analyzed (Supplementary Data [Supplementary-material supplementary-material-1]). The largest prophage identified had 48.1 kb and the smallest 22.4 kb, identified in the MV1022 and M62 strains, respectively (Supplementary Data [Supplementary-material supplementary-material-1]). The GC content of the identified prophages varied between 39.3 and 44.6% (Supplementary Data [Supplementary-material supplementary-material-1]).

**Figure 4:**
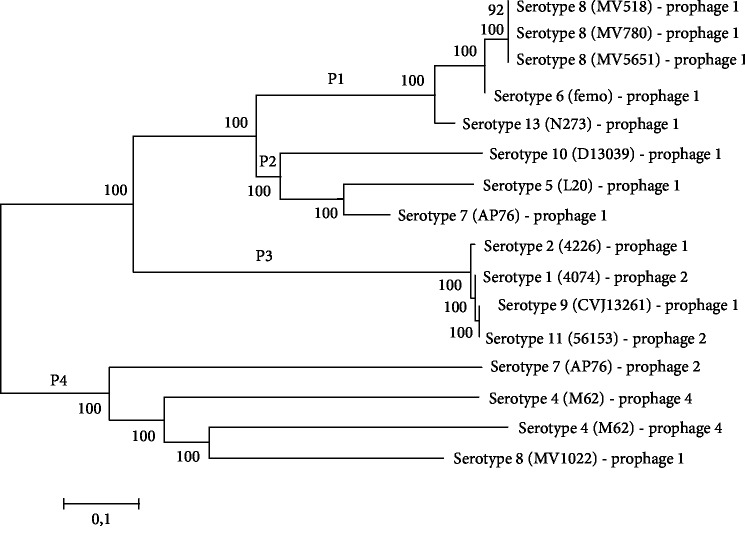
Phylogenetic relationships between sequences related to complete prophages found in *A. pleuropneumoniae*. The phylogenetic tree using the Neighbor-Joining method with 2000 bootstraps was generated by the MEGA 6 program after alignment by MAFFT and GBLOCKS. The scale is represented below, with 0.1 nucleotide substitutions per site.

The Neighbor-Joining method to cluster analysis between sequences similar to complete prophages allowed us to identify four different clusters (P1-P4) (Figure 4). Analysis of the complete prophages using the BLAST algorithm against sequences from the GenBank database showed that the sequences contained in the P3 cluster (prophage 2 (4074 strain), prophage 1 (4226 strain), prophage 1 (CVJ13261), and prophage 2 (56153 strain)), the M62 prophage 2, and the AP76 prophage 2 share over 70% identity and were found in genomes of *Haemophilus ducreyi* (AE017143.1), *Mannheimia haemolytica* (KP137440.1), and *Actinobacillus suis* (CP009159.1), respectively (Table 4). For the other 13 complete prophages, no significant identity or sequence coverage was found in GenBank.

**Figure 5:**
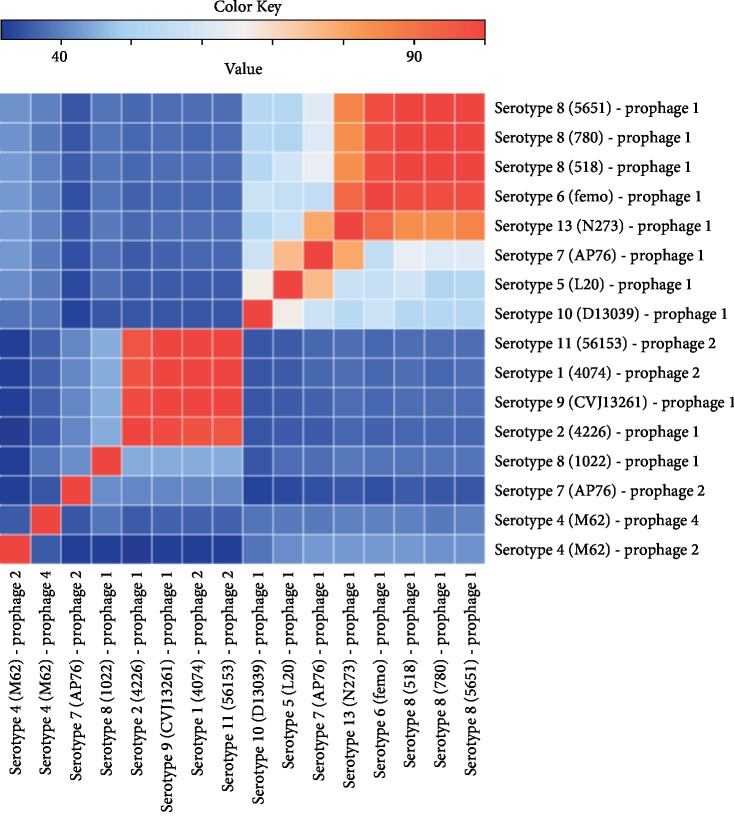
Heat map analysis from identity matrix generated by global alignment of 16 complete prophage genomes. The alignment was done using Clustal Omega, and the identity matrix generated was used to create the heat map by R software.

The heat map distribution showed a high identity among the prophage 1 from 518, 780, and 5651 (serotype 8) and femo (serotype 6) (Figure 5). This could also be observed among prophage 2 from 56153 (serotype 11) and 4074 (serotype 1) and prophage 1 from CVJ13261 (serotype 9) and 4226 (serotype 2). A considerable identity also was observed among prophage 1 from 518, 780, and 5651 (serotype 8), femo (serotype 6), and N273 (serotype 13). Similarly, it also was observed between N273 (serotype 13) and AP76 (serotype 7) and L20 (serotype 5) and AP76 (serotype 7).

## 4. Discussion

Analyses of GC content, codon usage, and amino acid use among the different *A. pleuropneumoniae* serotypes showed that they share a set of conserved CDS. The core portion of the genome that is well conserved among the serotypes also reinforces these results. Among the most commonly used amino acids are branched chain amino acids, such as leucine, isoleucine, and valine. These branched chain amino acids are required for the survival and virulence of *A. pleuropneumoniae* in swine, capable of synthesizing these amino acids critical for respiratory tract pathogens [39].

In the analyses of clusters from the set of CDS shared between serotypes, the pattern of clustering by similarity was compatible with the classification of serotypes into three virulence categories [16]: low, medium, and high virulence. We observed that serotype 8 shares a high number of protein-coding sequences with the serotypes characterized as having medium virulence, such as serotypes 2, 4, 6, 7, and 12. The characteristic of the serotypes considered as medium virulence category is associated with the persistence of the pathogen in the environment [8]. Additionally, a large sharing of CDS for serotype 8 proteins was observed in serotype 6, followed by serotype 3. As already reported in serotyping analyses, certain groups may cross-react and be mischaracterized. Serotypes 3, 6, and 8 of *A. pleuropneumoniae* in serotyping studies in North America constitute a single group, and discrimination of these three serotypes within this group is extremely difficult when using the antiserum technique [19].

In the COG analyses of the predicted amino acid sequences of *A. pleuropneumoniae* serotype 8, the core region is characterized by housekeeping genes. However, genes belonging to the core region may have differences in the level of DNA sequences. A number of genes classified as core like those encoding anaerobic glycerol-3-phosphate dehydrogenase subunit A (*glpA*), oxygen-independent coproporphyrinogen-III oxidase (*hemN*), heptosyltransferase family (*mutM*), tellurite resistance protein (*tehA*), sulfate transport system permease (*cysW*), thiazole biosynthesis protein (*thiH*), haloacid dehalogenase-like hydrolases (*had*) superfamily (*cof*), nucleoside diphosphate sugar epimerase, and oligopeptide transporter testify to positive selection in *A. pleuropneumoniae* [40]. In general, these genes are involved in the transporting of nutrients and cellular metabolism that show that *A. pleuropneumoniae* has responded to different environmental pressures.

In the core portion, we also found genes that, according to [41], have increased expression during the acute phase of natural infection of *A. pleuropneumoniae* in pigs. These genes were related, for example, to the assembly of curli fibers, important in the formation of biofilms [42]; to the maltose operon that may increase the competition capacity in some strains of pathogenic bacteria [43]; and to the ula operon involved with an ascorbate transport system under anaerobic conditions that can also be considered an important virulence factor for this species [41].

The accessory portion, comprising the shared portion and the differential, is characterized by genes that confer benefits to the microorganism under certain environmental conditions. The differential portion, as observed in *in silico* assays, has a strong relationship with HGT processes, containing sequence-encoding proteins common to plasmids and phages. This region can result in important adaptations, influencing the differentiated interaction of the pathogen with the host, as well as having an important role in the differentiation of serotypes and mechanisms of virulence. As regards the differential portion, few differential C were affiliated with the COG categories. As this part of the genome has not been studied in a judicious way, we have a great network of sequences that codify proteins characterized as hypothetical, which are not categorized in the COG analysis. Among the sequences found in the differential portion, two sequences relating to the LysR family are present in the reference genome of *A. pleuropneumoniae* serotype 8. LysR is a family of transcriptional regulators that regulate a diverse set of genes, including those involved in virulence, metabolism, quorum sensing, and motility [44]. This regulator has also been related to processes of regulation of genes that code for urease in pathogenic bacteria [45]. In the differential portion of reference genome serotype 8, sequences encoding tetracycline, florfenicol, and sulfonamide resistance proteins were also found. In previous studies, the presence of plasmids in *A. pleuropneumoniae* and other members of the family Pasteurellaceae, conferring resistance to florfenicol, chloramphenicol, and tetracycline, has been characterized [13, 46, 47].

Alignment of the *A. pleuropneumoniae* genomes allowed for the determination of gain/loss and sequence rearrangements between serotypes. In the serotype 7 strains, there are rearrangements relating to the presence of insertion elements, indicating a process of integration of moving elements. Transposable elements have the ability to move within the genome, and their insertion close to the coding regions may alter gene expression [48]. Transposable elements if present in multiple copies can serve as sites for ectopic recombination events in the genome. Finally, these elements can incorporate additional genes and subsequently act as vectors for these genes. Any change, insertion, deletion, or rearrangement that may occur in a genome may alter the expression of adjacent genes and generate a substantial impact on gene expression and pathogenesis of the microorganism [49, 50]. The alignment of the genomes also demonstrated the existence of variations between the serotypes analyzed. The differences in alignments largely correspond to sequences relating to the HGT process such as prophages. Prophages are phages that integrate into the bacterial genome, in which they play an important role in genomic diversity and may be related to the acquisition of virulence factors for the host cell [51]. The acquisition of foreign sequences to the genome may be related to the fact that *A. pleuropneumoniae* is capable of performing natural transformation and has different levels of competence among serotypes and even among isolates of the same serotype [11, 12].

The results observed in Figure 5 showed consistent relation with the phylogenetic analysis. It was possible to see because the prophage sequences that showed considerable or high identity are present at the same or close groups in the phylogenetic tree.

In this study, 16 putative sequences related to the complete prophages were identified in the 19 genomes analyzed. Similarity analysis of the complete prophage sequences found in *A. pleuropneumoniae* against the GenBank database identified high similarity and coverage with sequences present in the genomes of *A. suis*, *M. haemolytica*, and *H. ducreyi*, which may be related to HGT among species belonging to the family Pasteurellaceae. *A. suis* is commonly found in swine as tonsil commensal, but in the presence of unknown stimuli, it may invade the bloodstream, causing septicemia and sequelae, such as meningitis and arthritis, and even lead to the death of the host [52]. On the other hand, *M. haemolytica* is frequently involved in respiratory diseases in cattle [53] while *H. ducreyi* is a bacterium that causes soft chancre, a sexually transmitted disease in humans, and which has pigs as a model for studying the disease [54, 55]. Of the 6 sequences with high similarity and coverage identified in GenBank, only prophage 4 of the M62 strain had significant alignment correspondence with the phage sequence already described in the literature. This prophage was found in *M. haemolytica* and named vB_MhM_3927AP2 by the authors, being a phage belonging to the *Myoviridae* family [56]. The remaining 13 prophages have low identity and coverage in biological databases, suggesting that they may be phages unique to this species or not reported yet.

In conclusion, the genome of *A. pleuropneumoniae* serotype 8 is conserved in relation to the other serotypes, being more related to serotypes 3 and 6, which justifies the problems of serotyping to distinguish these three serotypes. We detected strong evidence of DNA sequence acquisition and recombination in the genomes of the different isolates/serotypes, and these differences were attributed to the presence of mobile genetic material, mainly prophages. In this study, we have identified 16 complete prophages, 6 of which may have suffered HGT among species belonging to the family Pasteurellaceae. However, the other prophages seem to be exclusive of *A. pleuropneumoniae* and not yet reported in the literature. Thus, prophages seem to play a key role in the restructuring of genomes and in the emergence of new strains of this pathogen.

## Figures and Tables

**Table 1:** *A. pleuropneumoniae* genomes used in this study.

Strain/serotype	Genome size (pb)	CDS	% GC	Accession code (WGS)	Reference
4074/1	2318649	2135	41.2	CP029003.1	Xu et al., [16]
4226/2	2314083	2228	41.2	ADXN00000000.1	Zhan et al., 2010
JL03, 3	2242062	2115	41.2	CP000687.1	Xu et al., 2008
M62/4	2260565	2186	41.2	ADOF00000000.1	Xu et al., [16]
L20/5b	2274482	2168	41.3	CP000569.1	Foote et al., 2008
Femo/6	2302700	2300	41.0	ADOG00000000.1	Xu et al., [16]
AP76/7	2345435	2234	41.2	CP001091.1	Linke et al., 2008
MV460/8	2213381	2116	41.1	JSVG00000000.1	Pereira et al., [23]
MV518/8	2275540	2189	41.1	JSVZ00000000.1	
MV597/8	2219395	2115	41.1	JSVX00000000.1	
MV780/8	2274000	2179	41.1	JSVV00000000.1	
MV1022/8	2262828	2179	41.1	JSVF00000000.1	
MV5651/8	2264279	2179	41.1	JSVY00000000.1	
MIDG2331/8	2337633	2235	41.1	LN908249.1	Bossé et al., [22]
CVJ13261/9	2256417	2163	41.2	ADOI00000000.1	Xu et al., [16]
D13039/10	2266276	2155	41.2	ADOJ00000000.1	
56153/11	2257884	2154	41.2	ADOK00000000.1	
1096/12	2185499	2082	41.2	ADOL00000000.1	
N273/13	2236660	2148	41.2	ADOM00000000.1	

**Table 2:** Coding sequences of *A. pleuropneumoniae* serotype 8.

COG	Description of COG classes	Core	Shared	Differential	Total
A	RNA modification and processing	1	0	0	1
C	Conversion and production of energy	117	9	0	126
D	Cycle control and cell division, chromosome partitioning	25	3	0	28
E	Amino acid metabolism and transport	158	10	1	169
F	Nucleotide metabolism and transport	60	2	0	62
G	Carbohydrate metabolism and transport	117	11	2	130
H	Coenzyme metabolism and transport	91	10	1	102
I	Lipid metabolism and transport	39	3	0	42
J	Translation, ribosomal structure, and biogenesis	153	7	2	162
K	Transcript	77	10	7	94
L	Replication, recombination, and repair	95	20	4	119
M	Biogenesis of cell wall, membrane, and envelope	127	21	0	148
N	Cellular motility	6	1	1	8
O	Posttranslational modification, protein turnover, and chaperones	96	6	0	102
P	Metabolism and transport of inorganic ions	104	24	0	128
Q	Biosynthesis of secondary metabolites, transport, and catabolism	8	5	0	13
T	Signal transduction mechanisms	31	1	0	32
U	Intracellular traffic, secretion, and vesicular transport	33	5	0	38
V	Defense mechanisms	20	18	0	38
R	Prediction of general functions	158	23	2	183
S	Unknown function	169	31	0	200
NC	Proteins not categorized on COG	116	216	95	427
	Total of affiliated proteins	1685	220	20	1925
	Total of serotype 8 proteins	1801	436	115	2352

**Table 3:** Characterization of protein groups in different serotypes of *A. pleuropneumoniae*.

Serotype	Total proteins	Core	Shared	Differential
		Proteins (%)	Proteins (%)	Proteins (%)
Serotype 1	2176	1765 (81.1)	404 (18.6)	7 (0.3)
Serotype 2	2064	1774 (86.0)	275 (13.3)	15 (0.7)
Serotype 3	2026	1756 (86.7)	260 (12.8)	10 (0.5)
Serotype 4	2219	1790 (80.7)	325 (14.7)	104 (4.7)
Serotype 5b	2004	1765 (88.1)	208 (10.4)	31 (1.6)
Serotype 6	2211	1768 (80.0)	384 (17.4)	59 (2.7)
Serotype 7	2113	1774 (84.0)	327 (15.5)	12 (0.6)
Serotype 8^∗^	2352	1801 (76.6)	436 (18.5)	115 (4.9)
Serotype 9	2197	1779 (81.0)	416 (18.9)	2 (0.1)
Serotype 10	2170	1774 (82.0)	321 (14.8)	75 (3.5)
Serotype 11	2184	1767 (81.0)	414 (19.0)	3 (0.1)
Serotype 12	2081	1771 (85.1)	294 (14.1)	16 (0.8)
Serotype 13	2145	1760 (82.1)	381 (17.8)	4 (0.2)

^∗^Reference genome represents all the sequences encoding the seven clinical isolate genomes previously sequenced.

**Table 4:** Identification of complete prophage sequences of *A. pleuropneumoniae*.

Strain/serotype	Prophage sequence identified	Organism/accession	ID %	Coverage	*E* value
4074/1	2	*Haemophilus ducreyi*/AE017143.1	97	85	0.0
4226/2	1	*Haemophilus ducreyi*/AE017143.1	98	95	0.0
M62/4	2	*Actinobacillus equuli*/CP007715.1	94	7	0.0
M62/4	4	*Mannheimia haemolytica*/KP137440.1	86	76	0.0
L20/5b	1	*Mannheimia* sp./CP006942.1	87	23	0.0
Femo/6	1	*Mannheimia haemolytica*/CP004753.1	89	25	0.0
AP76/7	1	*Mannheimia* sp./CP006942.1	89	26	0.0
	2	*Actinobacillus suis*/CP009159.1	89	86	0.0
MV518/8	1	*Mannheimia haemolytica*/CP004753.1	86	12	0.0
MV780/8	1	*Mannheimia haemolytica*/CP004753.1	86	14	0.0
MV1022/8	1	*Mannheimia haemolytica*/CP004753.1	86	12	0.0
MV5651/8	1	*Mannheimia haemolytica*/CP004753.1	86	15	0.0
CVJ13261/9	1	*Haemophilus ducreyi*/AE017143.1	97	89	0.0
D13039/10	1	*Mannheimia haemolytica*/CP004753.1	83	23	0.0
56153/11	2	*Haemophilus ducreyi*/AE017143.1	97	85	0.0
N273/13	1	*Mannheimia haemolytica*/CP004753.1	86	14	0.0

## Data Availability

The data that support the results of this study are available in databases described in the manuscript and from the corresponding authors upon request.

## Supplementary Materials

Supplementary Materials Supplementary Data Table 1: differential protein sequences found in *A. pleuropneumoniae* serotype 8. Supplementary Data Table 2: putative prophage sequences identified in the *A. pleuropneumoniae* genomes analyzed.
