# Supplementary material for: Comparative Genomics of Actinobacillus pleuropneumoniae Serotype 8 Reveals the Importance of Prophages in the Genetic Variability of the Species
Source: Int J Genomics. 2020 Feb 18;2020:9354204. doi: 10.1155/2020/9354204 (PMC7049842; doi:10.1155/2020/9354204)
Supplement: Supplementary Materials — Supplementary Data Table 1: differential protein sequences found in A. pleuropneumoniae serotype 8. Supplementary Data Table 2: putative prophage sequences identified in the A. pleuropneumoniae genomes analyzed. [file 9354204.f1.docx]

**Supplementary information**

**International Journal of Genomics**

**Comparative genomics of *Actinobacillus pleuropneumoniae* serotype 8 reveals the importance of prophages in the genetic variability of the species**

Isabelle Gonçalves de Oliveira Prado^1^, Giarlã Cunha da Silva^1^, Josicelli Souza Crispim^1^, Pedro Marcus Pereira Vidigal^2^, Moysés Nascimento^3^, Mateus Ferreira Santana^1^, Denise Mara Soares Bazzolli^1*^.

^1^ Laboratório de Genética Molecular de Bactérias/ Bioagro - Departamento de Microbiologia, Universidade Federal de Viçosa, Viçosa 36570-900, Brazil.

^2^ Núcleo de Biomoléculas, Universidade Federal de Viçosa, Viçosa, Brazil.

^3^ Departamento de Estatística, Universidade Federal de Viçosa, Viçosa, Brazil.

***Corresponding author:**

Denise Mara Soares Bazzolli (dbazzolli@ufv.br); phone: +55 (31) 3612-2454

**Table S1** Differential proteins sequences found in *A. pleuropneumoniae* serotype 8

| **Identification** | **UNIPROT** | **Description of the Sequences** | **COG^a^** |
| --- | --- | --- | --- |
| App8_1475 | A0A0S4QBQ6 | Acetyltransferase | R |
| App8_1176 | A0A0S4QBH9 | AntA/AntBantirepressor | K |
| App8_1295 | A0A0S4QBY2 | Antibiotic biosynthesis monooxygenase | R |
| App8_2233 | D9PEA9 | Bacteriophage CII protein | - |
| App8_2238 | [A0A0E3ZR94](http://www.uniprot.org/uniprot/A0A0E3ZR94) | DNA or RNA helicase of superfamily II | K |
| App8_2263 | B3H0W4 | Cro repressor | K |
| App8_1256 | A0A0S4QAY3 | DNA primase TraC | J |
| App8_1297 | A0A0S4QB64 | DNA-binding transcriptional regulator LysR | K |
| App8_1299 | A0A0S4QBD3 | DNA repair protein | J |
| App8_2313 | A0A0G3Y3F6 | Florfenicol/chloramphenicol resistance protein | G |
| App8_1246 | A0A0S4QB36 | Integrase/recombinaseXerC | L |
| App8_2262 | D9PEB1 | LexA family repressor/S24 family protease | K |
| App8_2314 | E0EKB4 | LysR family transcriptional regulator | K |
| App8_2318 | Q3HYC8 | MobC | - |
| App8_574 | A0A0S4Q8V1 | Phage X family protein | - |
| App8_1278 | A0A0S4QB66 | Plasmid protein of unknown function (plasmid_RAQPRD) | - |
| App8_710 | A0A0S4Q9A0 | Putative HTH-type transcriptional regulator | K |
| App8_714 | A0A0S4Q9B2 | Recombination protein F | L |
| App8_2316 | Q3HYC5 | Plasmid replication proteins, Rep | L |
| App8_1296 | A0A0S4QBA4 | Sodium/glutamate symporter | E |
| App8_2308 | Q3B8S2 | TetH | G |
| App8_2309 | Q3B8S3 | Tetracycline repressor protein TetR | H |
| App8_1253 | A0A0S4QDE8 | Transposon gamma-delta resolvase | L |
| App8_1262 | A0A0S4QB51 | TraU protein | - |
| App8_1286 | A0A0S4QBF4 | TraX protein | - |
| App8_577 | A0A0S4QA99 | Type II secretion system protein D | N |
| App8_2356 | A0A0S4QAU0 | Ash protein family protein | - |
| App8_2373 | D9P9R5 | Hypothetical protein | - |
| App8_2204 | E0FQZ0 | Hypothetical protein | - |
| App8_2239 | [A0A0E3ZQL4](http://www.uniprot.org/uniprot/A0A0E3ZQL4) | Hypothetical protein | - |
| App8_2264 | [A6VNN3](http://www.uniprot.org/uniprot/A6VNN3) | Hypothetical protein | - |
| App8_2268 | A0A011LWF5 | Hypothetical protein | - |
| App8_2272 | No hit^b^ | Hypothetical protein | - |
| App8_2310 | R9UPZ9 | Hypothetical protein | - |
| App8_2315 | E0FQZ0 | Hypothetical protein | - |
| App8_2267 | D9PEA3 | Hypothetical protein | - |
| App8_2271 | E0E6Z6 | Hypothetical protein | - |
| App8_2281 | D9PEJ6 | Hypothetical protein | - |
| App8_2336 | A9HSL6 | Hypothetical protein | - |
| App8_2359 | Q4W2Q8 | Hypothetical protein | - |
| App8_2203 | [E9ND26](http://www.uniprot.org/uniprot/E9ND26) | Hypothetical protein | - |
| App8_453 | A0A0S4Q8W0 | Hypothetical protein | - |
| App8_569 | A0A0S4Q9Q3 | Hypothetical protein | - |
| App8_570 | A0A0S4Q8T1 | Hypothetical protein | - |
| App8_571 | A0A0S4Q971 | Hypothetical protein | - |
| App8_572 | A0A0S4Q8U2 | Hypothetical protein | - |
| App8_573 | A0A0S4Q8S2 | Hypothetical protein | - |
| App8_575 | A0A0S4QBE1 | Hypothetical protein | - |
| App8_576 | A0A0S4Q920 | Hypothetical protein | - |
| App8_578 | A0A0S4Q8R1 | Hypothetical protein | - |
| App8_579 | A0A0S4Q8P5 | Hypothetical protein | - |
| App8_1067 | A0A0S4QB97 | Hypothetical protein | - |
| App8_1177 | A0A0S4QAU7 | Hypothetical protein | - |
| App8_1178 | A0A0S4QAW4 | Hypothetical protein | - |
| App8_1248 | A0A0S4QB60 | Hypothetical protein | - |
| App8_1249 | A0A0S4QB27 | Hypothetical protein | - |
| App8_1250 | A0A0S4QB44 | Hypothetical protein | - |
| App8_1251 | A0A0S4QDP9 | Hypothetical protein | - |
| App8_1252 | A0A0S4QB87 | Hypothetical protein | - |
| App8_1254 | A0A0S4QBA8 | Hypothetical protein | - |
| App8_1257 | A0A0S4QDC9 | Hypothetical protein | - |
| App8_1258 | A0A0S4QAY4 | Hypothetical protein | - |
| App8_1259 | A0A0S4QBS1 | Hypothetical protein | - |
| App8_1260 | A0A0S4QCA7 | Hypothetical protein | - |
| App8_1261 | A0A0S4QAY1 | Hypothetical protein | - |
| App8_1263 | A0A0S4QBV7 | Hypothetical protein | - |
| App8_1264 | A0A0S4QB74 | Hypothetical protein | - |
| App8_1265 | A0A0S4QB40 | Hypothetical protein | - |
| App8_1266 | A0A0S4QB59 | Hypothetical protein | - |
| App8_1267 | A0A0S4QDW8 | Hypothetical protein | - |
| App8_1268 | A0A0S4QB99 | Hypothetical protein | - |
| App8_1269 | A0A0S4QDG8 | Hypothetical protein | - |
| App8_1271 | A0A0S4QDH9 | Hypothetical protein | - |
| App8_1272 | A0A0S4QB00 | Hypothetical protein | - |
| App8_1273 | A0A0S4QDE3 | Hypothetical protein | - |
| App8_1274 | A0A0S4QAZ9 | Hypothetical protein | - |
| App8_1275 | A0A0S4QBT4 | Hypothetical protein | - |
| App8_1276 | A0A0S4QCB9 | Hypothetical protein | - |
| App8_1277 | A0A0S4QAZ7 | Hypothetical protein | - |
| App8_1279 | A0A0S4QBX0 | Hypothetical protein | - |
| App8_1280 | A0A0S4QB90 | Hypothetical protein | - |
| App8_1282 | A0A0S4QB75 | Hypothetical protein | - |
| App8_1283 | A0A0S4QDY1 | Hypothetical protein | - |
| App8_1284 | A0A0S4QBB6 | Hypothetical protein | - |
| App8_1285 | A0A0S4QDI1 | Hypothetical protein | - |
| App8_1287 | A0A0S4QDJ4 | Hypothetical protein | - |
| App8_1294 | A0A0S4QB88 | Hypothetical protein | - |
| App8_1298 | A0A0S4QDZ8 | Hypothetical protein | - |
| App8_1301 | A0A0S4QBG6 | Hypothetical protein | - |
| App8_1303 | A0A0S4QB32 | Hypothetical protein | - |
| App8_1304 | A0A0S4QDH3 | Hypothetical protein | - |
| App8_1306 | A0A0S4QBW0 | Hypothetical protein | - |
| App8_1307 | A0A0S4QCE2 | Hypothetical protein | - |
| App8_1308 | A0A0S4QB19 | Hypothetical protein | - |
| App8_1309 | A0A0S4QBA2 | Hypothetical protein | - |
| App8_1310 | A0A0S4QBZ5 | Hypothetical protein | - |
| App8_1472 | A0A0S4QCM0 | Hypothetical protein | - |
| App8_1474 | A0A0S4QBW1 | Hypothetical protein | - |
| App8_1543 | A0A0S4QC22 | Hypothetical protein | - |
| App8_1544 | A0A0S4QCV2 | Hypothetical protein | - |
| App8_1689 | A0A0S4QCL8 | Hypothetical protein | - |
| App8_1700 | A0A0S4QDL4 | Hypothetical protein | - |
| App8_1701 | A0A0S4QC39 | Hypothetical protein | - |
| App8_1704 | A0A0S4QCC7 | Hypothetical protein | - |
| App8_1981 | A0A0S4QD57 | Hypothetical protein | - |
| App8_2213 | No hit | Hypothetical protein | - |
| App8_2012 | No hit | Hypothetical protein | - |
| App8_2013 | [A7RPE6](http://www.uniprot.org/uniprot/A7RPE6) | Hypothetical protein | - |
| App8_2127 | [E1W2I6](http://www.uniprot.org/uniprot/E1W2I6) | Hypothetical protein | - |
| App8_2202 | No hit | Hypothetical protein | - |
| App8_2259 | No hit | Hypothetical protein | - |
| App8_2305 | No hit | Hypothetical protein | - |
| App8_2311 | Q629K6 | Hypothetical protein | - |
| App8_2317 | A0A0H4H1T6 | Hypothetical protein | - |
| App8_2234 | D9PEA7 | Hypothetical protein | - |

^a^ R: Prediction of general functions, K: Transcript, (-): Proteins not categorized on COG, J: Translation, ribosomal structure and biogenesis, G: Carbohydrates metabolism and transport, L: Replication, recombination and repair, E: Amino acid metabolism and transport, H: Coenzymes metabolism and transport, N: Cellular motility.
^b^ No hit: proteins that are not assigned to a defined COG category.

**Table S2** Prophages sequences putative identified in the *A. pleuropneumoniae* genomes analyzed

| **N°** | **Strains/**  **Serotype** | **Prophage sequence identified** | **Contig: localization** | **Length (Kb)** | **Classification** | **% GC** | **CDS** | **% Genome** |
| --- | --- | --- | --- | --- | --- | --- | --- | --- |
| 1 | 4074/1 | 1 | 37: 36042-63750 | 27.7 | Incomplete | 39.6 | 32 | 3.0 |
| 2 |  | 2 | 29: 227896-269422 | 41.5 | Complete | 41.0 | 58 |  |
| 3 | 4226/2 | 1 | 5: 87591-123663 | 36 | Complete | 41.6 | 45 | 1.5 |
| 4 | JL03/3 | 1 | 247542-251087 | 3.5 | Incomplete | 39.3 | 9 | 1.9 |
| 5 |  | 2 | 1225994-1232700 | 6.7 | Incomplete | 39.7 | 8 |  |
| 6 | M62/4 | 1 | 37: 1-13914 | 13.9 | Incomplete | 39.5 | 30 | 3.3 |
| 7 |  | 2 | 38: 9940-35121 | 25.1 | Complete | 43.1 | 29 |  |
| 8 |  | 3 | 88: 3-14096 | 14 | Incomplete | 42.3 | 29 |  |
| 9 |  | 4 | 103: 22605-45103 | 22.4 | Complete | 44.6 | 26 |  |
| 10 | L20/5b | 1 | 557157-599435 | 42.2 | Complete | 40.4 | 58 | 1.8 |
| 11 | Femo/6 | 1 | 22: 571-23431 | 22.8 | Complete | 42.8 | 22 | 3.0 |
| 12 |  | 2 | 26: 105577-138426 | 32.8 | Questionable | 40.0 | 28 |  |
| 13 |  | 3 | 40: 1-15121 | 15.1 | Incomplete | 39.8 | 31 |  |
| 14 | AP76/7 | 1 | 517113-563510 | 46.3 | Complete | 41.5 | 67 | 3.4 |
| 15 |  | 2 | 887488-920941 | 33.4 | Complete | 40.0 | 31 |  |
| 16 | MV460/8 | 1 | 19: 31463-38166 | 6.7 | Incomplete | 39.7 | 9 | 0.3 |
| 17 | MV518/8 | 1 | 7: 47533-95598 | 48 | Complete | 40.9 | 73 | 2.1 |
| 18 | MV597/8 | 1 | 14: 122700-130010 | 7.3 | Incomplete | 41.9 | 9 | 0.6 |
| 19 |  | 2 | 19: 31466-38169 | 6.7 | Incomplete | 39.7 | 9 |  |
| 20 | MV780/8 | 1 | 10: 44-41676 | 41.6 | Complete | 41.4 | 57 | 1.8 |
| 21 | MV1022/8 | 1 | 42: 57-48228 | 48.1 | Complete | 40.9 | 72 | 2.1 |
| 22 | MV5651/8 | 1 | 8: 2844-41377 | 38.5 | Complete | 41.3 | 52 | 1.7 |
| 23 | MIDG2331/8 | 1 | 1237208-1243914 | 6.7 | Incomplete | 39.7 | 8 | 0.3 |
| 24 | CVJ13261/9 | 1 | 24: 28089-66677 | 38.5 | Complete | 41.3 | 54 | 1.7 |
| 25 | D13039/10 | 1 | 21: 18252-45077 | 26.8 | Complete | 41.2 | 34 | 1.2 |
| 26 | 56153/11 | 1 | 28: 36562-64275 | 27.7 | Incomplete | 39.6 | 31 | 3.0 |
| 27 |  | 2 | 20: 182312-223849 | 41.5 | Complete | 41.0 | 57 |  |
| 28 | 1096/12 | 1 | 76: 45755-78609 | 32.8 | Questionable | 40.0 | 26 | 1.5 |
| 29 | N273/13 | 1 | 04: 121721-168475 | 46.7 | Complete | 41.2 | 65 | 3.5 |
| 30 |  | 2 | 230: 43788-77241 | 33.4 | Questionable | 40.0 | 29 |  |
